# Evidence for impaired chronotropic responses to and recovery from 6‐minute walk test in women with post‐acute COVID‐19 syndrome

**DOI:** 10.1113/EP089965

**Published:** 2021-11-17

**Authors:** Marissa N. Baranauskas, Stephen J. Carter

**Affiliations:** ^1^ Department of Kinesiology School of Public Health – Bloomington Indiana University Bloomington Indiana USA; ^2^ Indiana University Melvin and Bren Simon Comprehensive Cancer Center Indiana University School of Medicine Indianapolis Indiana USA

**Keywords:** 6‐minute walk test, exercise, heart rate recovery

## Abstract

**New Findings:**

- **What is the central question of this study?**
  Are chronotropic responses to a 6‐minute walk test different in women with post‐acute coronavirus disease 2019 (COVID‐19) syndrome compared with control subjects?
- **What is the main finding and its importance?**
  Compared with control subjects, the increase in heart rate was attenuated and recovery delayed after a 6‐minute walk test in participants after infection with severe acute respiratory syndrome coronavirus 2 (SARS‐CoV‐2). Women reporting specific symptoms at time of testing had greater impairments compared with control subjects and SARS‐CoV‐2 participants not actively experiencing these symptoms. Such alterations have potential to constrain not only exercise tolerance but also participation in free‐living physical activity in women during post‐acute recovery from COVID‐19.

**Abstract:**

The short‐term cardiopulmonary manifestations of severe acute respiratory syndrome coronavirus 2 (SARS‐CoV‐2) are well defined. However, the implications of cardiopulmonary sequelae, persisting beyond acute illness, on physical function are largely unknown. Herein, we characterized heart rate responses to and recovery from a 6‐minute walk test (6MWT) in women ∼3 months after mild‐to‐moderate SARS‐CoV‐2 infection compared with non‐infected control subjects. Forty‐five women (*n* = 29 SARS‐CoV‐2; *n* = 16 controls; age = 56 ± 11 years; body mass index = 25.8 ± 6.0 kg/m^2^) completed pulmonary function testing and a 6MWT. The SARS‐CoV‐2 participants demonstrated reduced total lung capacity (84 ± 8 vs. 93 ± 13%; *P* = 0.006), vital capacity (87 ± 10 vs. 93 ± 10%; *P* = 0.040), functional residual capacity (75 ± 16 vs. 88 ± 16%; *P* = 0.006) and residual volume (76 ± 18 vs. 93 ± 22%; *P* = 0.001) compared with control subjects. No between‐group differences were observed in 6MWT distance (*P* = 0.194); however, the increase in heart rate with exertion was attenuated among SARS‐CoV‐2 participants compared with control subjects (+52 ± 20 vs. +65 ± 18 beats/min; *P* = 0.029). The decrease in heart rate was also delayed for minutes 1–5 of recovery among SARS‐CoV‐2 participants (all *P *< 0.05). Women reporting specific symptoms at the time of testing had greater impairments compared with control subjects and SARS‐CoV‐2 participants not actively experiencing these symptoms. Our findings provide evidence for marked differences in chronotropic responses to and recovery from a 6MWT in women several months after acute SARS‐CoV‐2 infection.

## 1 INTRODUCTION

More than 225 million individuals have contracted acute respiratory syndrome coronavirus 2 (SARS‐CoV‐2), the virus responsible for the coronavirus disease 2019 (COVID‐19) pandemic. With progressive vaccination efforts, case incidence rates are declining; however, the functional consequences of cardiopulmonary sequelae persisting for longer than 3–4 weeks after the onset of initial symptoms, termed ‘post‐acute COVID‐19 syndrome’ (Nalbandian et al., 2021), remain unclear. Longitudinal monitoring of hospitalized SARS‐CoV‐2 patients has revealed restrictive defects and/or diffusion impairments in > 50% of patients in the months following discharge (Bellan et al., 2021; Orzes et al., 2021; Safont et al., 2021). Although male sex is recognized as a predictor for increased COVID‐19 disease severity and mortality (Peckham et al., 2020), female sex has been associated with a greater risk for persistent diffusion impairments months into recovery (Bellan et al., 2021; Huang et al., 2021; Safont et al., 2021). Additional data indicate that, after SARS‐CoV‐2 infection, women commonly report declining physical health or fatigue (Xiong et al., 2021) and are three to four times more likely to seek treatment for chronic symptoms (Davido et al., 2020; Vanichkachorn et al., 2021). In particular, persistent cardiopulmonary abnormalities following a SARS‐CoV‐2 infection may reduce overall exercise tolerance in women.

The 6‐minute walk test (6MWT) is a widely used clinical assessment of functional exercise capacity (Lancaster, 2018); the heart rate (HR) response to this test is a strong independent predictor of daily physical activity (Morita et al., 2018) and mortality (Holland et al., 2013; Swigris et al., 2009) in pulmonary disease patients. However, it remains unclear whether chronotropic responses to a 6MWT are altered among women during post‐acute recovery from COVID‐19. Therefore, in the present study we sought to characterize HR responses to and recovery from a 6MWT in women ≥ 4 weeks after a diagnosed SARS‐CoV‐2 infection in comparison to control women (i.e., no infection) matched for age and body mass index (BMI).

## 2 METHODS

### 2.1 Ethical approval

This study was approved by the Indiana University Institutional Review Board (2004439367) and performed in accordance with the latest revision of the *Declaration of Helsinki*, except for registration in a database. All participants provided written informed consent before enrolment.

### 2.2 Participants

Women ≥ 4 weeks after a positive diagnostic laboratory test for SARS‐CoV‐2 and women who had never tested positive for SARS‐CoV‐2 (controls) were enrolled in this case–control study. Participants were recruited from the surrounding community within a 185‐kilometer radius of Bloomington, IN, USA. Those with a documented history of major pulmonary disease (e.g., chronic obstructive pulmonary disease, cystic fibrosis or emphysema), major cardiovascular disease (e.g., congestive heart failure, stroke, myocardial infarction or coronary artery disease) and/or reported use of smoking/tobacco products within the last 6 months were excluded from participation.

### 2.3 Study design

After completing an initial telephone screening, participants were invited to the Human Performance Laboratory at Indiana University – Bloomington to complete a comprehensive pulmonary function testing assessment and 6MWT. Upon arrival, body weight and body composition were measured using a digital scale (MC‐790U, Tanita Corporation, Tokyo, Japan) and dual‐energy X‐ray absorptiometry (iDXA; Lunar, GE Healthcare, Chicago, IL, USA), respectively. Pulmonary function tests were performed using a Vmax Encore system (Vyaire Medical, Mettawa, IL, USA) to measure forced vital capacity (FVC), forced expiratory volume in 1 s (FEV_1_), forced expiratory flow rate between 25 and 75% of expired vital capacity (FEF_25–75%_), diffusing capacity of the lung for carbon monoxide (DLCO), alveolar volume (VA), total lung capacity (TLC), functional residual capacity (FRC), vital capacity (VC), inspiratory capacity (IC) and residual volume (RV) in accordance with the American Thoracic Society/European Respiratory Society (ATS/ETS) guidelines (Graham et al., 2017, 2019; Wanger et al., 2005). Pulmonary function test values are reported as a percentage of the predicted reference derived from the most recent Global Lung Initiative (GLI), and lower limits of normal (LLN) were defined as values below a *z*‐score of −1.96 (Quanjer et al., 2012).

After 10 min of seated rest, participants completed a 6MWT consistent with ATS guidelines (‘ATS statement: guidelines for the six‐minute walk test’, 2002). Before walking, resting HR (Polar Electro, Kempele, Finland), peripheral oxyhaemoglobin ($S_{pO_{2}}$) and carboxyhaemoglobin saturation (SpCO; Pronto, Masimo, Irvine, CA, USA), brachial blood pressure (BP; CT40, SunTech Medical, Morrisville, SC, USA) and ratings of perceived dyspnoea (RPD; Borg, 1998; CR10) were recorded. Measures of HR, $S_{pO_{2}}$, RPD and ratings of perceived exertion using a 100 mm visual analog scale ranging from ‘no exertion’ to ‘maximal exertion’ (RPE_VAS_) were assessed immediately post‐6MWT and throughout 5 min of standing recovery. Heart rate recovery (HRR) was calculated as the difference in HR immediately post‐6MWT in comparison to each minute of recovery (HRR_1min_, HRR_2min_, etc.). The 6MWT distance was normalized to a reference sample of > 200 women (median age = 58 years and mean BMI = 27.6 ± 4 kg/m^2^) using a prediction equation from Casanova et al. (2011) (%Pred 6MWT distance).

### 2.4 Statistical analyses

Analyses were performed using SPSS v.27.0 (IBM, Armonk, NY, USA). Data homogeneity was assessed using Levene's test and normality of distributions using the Shapiro–Wilks test. Student's unpaired *t*‐tests and Mann–Whitney *U*‐tests were used to compare participant characteristics, pulmonary function tests, resting HR, BP, $S_{pO_{2}}$, SpCO and RPD, post‐6MWT HRR, CR, $S_{pO_{2}}$, SpCO, RPD and RPE_VAS_, 6MWT distance and %Pred 6MWT distance between SARS‐CoV‐2 participants and control subjects for normally distributed and non‐normally distributed variables, respectively. MANCOVAs with Bonferroni corrections were used to compare HRR between groups after adjustment for the percentage of predicted 6MWT distance, resting HR, age and BMI. One‐way ANOVAs were used to compare %Pred 6MWT distance and HR responses between SARS‐CoV‐2 participants stratified by specific symptoms reported at the time of testing (i.e., symptomatic vs. asymptomatic) and control subjects. Associations between groups (SARS‐CoV‐2 vs. control) and pulmonary function tests below the LLN in addition to co‐morbidities were evaluated using Fisher's exact tests. Normally distributed data are presented as means ± SD and non‐normally distributed data as medians ± interquartile range. Statistical significance for all tests were set a priori and defined as a two‐sided *P*‐value ≤ 0.05.

## 3 RESULTS

### 3.1 Participant characteristics

**FIGURE 1:**
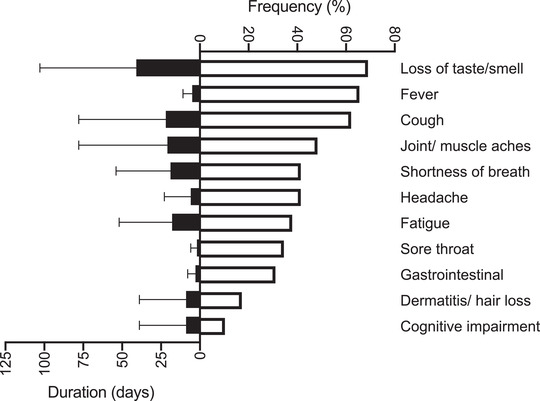
Coronavirus disease 2019 (COVID‐19) symptom inventory. Frequency and mean (SD) duration (in days) of symptoms reported by the severe acute respiratory syndrome coronavirus 2 (SARS‐CoV‐2) group (*n *= 29) at illness onset

Participant characteristics are provided in Table 1. The median time from positive diagnosis to enrolment was 94 ± 43 days. At the time of testing, 17 of 29 participants (59%) were symptomatic for cough, shortness of breath, fatigue, loss of taste/smell, joint/muscle aches and/or dermatitis/hair loss. One participant was briefly hospitalized (< 24 h) at the onset of illness prior to enrolment for complaints of chest and neck pain and/or pressure. No other participants were hospitalized. Consistent with definitions provided by the National Institutes of Health, all SARS‐CoV‐2 participants met criteria for mild‐to‐moderate illness severity (National Institutes of Health, 2021). Figure 1 illustrates the frequency and duration of symptoms reported by the SARS‐CoV‐2 group from the onset of illness.

**TABLE 1:** Characteristics of participants in the severe acute respiratory syndrome coronavirus 2 and control groups at rest and after 6‐minute walk test

**Variable**	**SARS‐CoV‐2 (*n* = 29)**	**Control (*n *= 16)**	***P*‐value**
Age, years	54 ± 10	58 ± 11	0.324
Co‐morbidities, *n* (%)			
Hypertension	7 (24)	3 (19)	1.000
Hypercholesterolaemia	2 (7)	3 (19)	0.330
Hypothyroidism	5 (17)	2 (13)	1.000
Rheumatoid arthritis	2 (7)	0 (0)	0.531
Asthma	2 (7)	2 (13)	0.608
History of cancer	1 (3)	0 (0)	1.000
Anxiety/depression	8 (28)	1 (6)	0.071
BMI, kg/m^2^	25.6 ± 5.4	26.7 ± 4.8	0.740
iDXA BF, %	38.7 ± 8.2	39.0 ± 8.9	0.895
Pre‐6MWT			
SBP, mmHg	128 ± 4	128 ± 11	0.962
DBP, mmHg	79 ± 10	81 ± 9	0.575
HR, beats/min	73 ± 12	70 ± 9	0.389
RPD, Borg CR10	0 ± 0	0 ± 0	0.928
$S_{pO_{2}}$, %	98 ± 3	99 ± 2	0.457
SpCO, %	0 ± 1	0 ± 1	0.901
Post‐6MWT			
6MWT distance, m	539 ± 101	579 ± 96	0.194
%Pred 6MWT distance, %	97 ± 17	101 ± 11	0.454
RPE_VAS_, 0–100 mm	28 ± 14	35 ± 19	0.218
HR, beats/min	124 ± 22	135 ± 20	0.092
RPD, Borg CR10	2.5 ± 1.9	2.8 ± 1.5	0.301
$S_{pO_{2}}$, %	99 ± 3	99 ± 4	0.640
SpCO, %	0 ± 0	0 ± 0	0.868

Abbreviations: BMI, body mass index; DBP, diastolic blood pressure; HR, heart rate; iDXA BF, body fat percentage measured using dual‐energy X‐ray absorptiometry; RPD, ratings of perceived dyspnoea using the Borg CR10 scale; RPE_VAS_, ratings of perceived exertion using a 100 mm visual analog scale ranging from ‘no exertion’ to ‘maximal exertion’; SARS‐CoV‐2, severe acute respiratory syndrome coronavirus 2; SBP, systolic blood pressure; SpCO, peripheral carboxyhaemoglobin saturation; $S_{pO_{2}}$, peripheral oxyhaemoglobin saturation; 6MWT, 6‐minute walk test; %Pred 6MWT distance, distance travelled during the 6MWT normalized to a reference sample using predictive equations provided by Casanova et al. (2011). *Note*. One subject in the SARS‐CoV‐2 group did not complete the 6MWT and is therefore not included in 6MWT variables (*n* = 28). Non‐normally distributed data (age for control subjects; BMI and post‐6MWT RPD for SARS‐CoV‐2 subjects; and rested and post‐6MWT $S_{pO_{2}}$, SpCO and RPD for both groups) are displayed as the median ± interquartile range. All other data are normally distributed and displayed as the mean ± SD or frequency [*n* (%)]. *P* > 0.05 between SARS‐CoV‐2 and control groups for all comparisons.

### 3.2 No between‐group difference in heart rate, blood pressure, oxyhaemoglobin saturation, carboxyhaemoglobin saturation or ratings of perceived dyspnoea measured at rest and immediately post‐6MWT

Resting and post‐6MWT variables are provided in Table 1. There were no differences in resting HR, systolic BP, diastolic BP, $S_{pO_{2}}$, SpCO or RPD between the SARS‐CoV‐2 group and control subjets. Additionally, there were no between‐group differences in post‐6MWT HR, $S_{pO_{2}}$, SpCO or RPD.

### 3.3 Between‐group differences detected in chronotropic response to and recovery from the 6MWT

**FIGURE 2:**
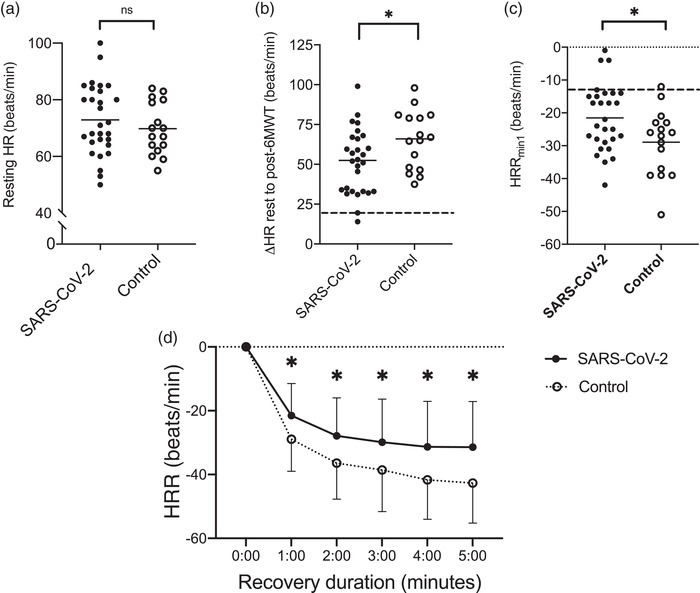
Heart rate (HR) responses to the 6‐minute walk test (6MWT). (a) Resting HR. (b) The chronotropic response (postexercise HR minus resting HR) to the 6MWT. The dashed line indicates the threshold (≤ +20 beats/min) predictive of mortality in interstitial lung disease (Holland et al., 2013). (c) Individual data points for heart rate recovery (HRR) taken immediately postexercise compared with the first minute of recovery after the 6MWT (HRR_1min_). Note that a threshold (≤ −13 beats/min) is predictive of mortality in idiopathic pulmonary fibrosis patients, as indicated by the dashed line (Swigris et al., 2009). (d) Heart rate recovery at each minute during 5 min of standing recovery after the 6MWT. One participant in the severe acute respiratory syndrome coronavirus 2 (SARS‐CoV‐2) group opted not to participate in the 6MWT and is therefore not included in analyses (*n* = 28). Data are presented as the mean ± SD. ^*^ *P *≤ 0.05 between SARS‐CoV‐2 and control groups

Figure 2a shows there are no between‐group differences in resting heart rate. Figure 2b illustrates that the ΔHR from rest to post‐6MWT was attenuated (i.e., increased less) among SARS‐CoV‐2 participants (+52 ± 20 beats/min) compared with control subjects (+65 ± 18 beats/min; *P* = 0.029). Additionally, Figure 2c, d demonstrates that HRR was delayed (i.e., decreased less) among SARS‐CoV‐2 throughout each minute of recovery despite a similar absolute and percent of predicted distance travelled. Between‐group differences in RPE_VAS_ were not observed (Table 1). Table 2 shows that differences in HRR persisted even after separate adjustment for %Pred 6MWT distance, resting HR, BMI and age.

**TABLE 2:** Group differences in HRR after the 6‐minute walk test adjusted for %Pred 6MWT distance, resting heart rate, body mass index and age

**Variable**	**SARS‐CoV‐2 (*n* = 28)**	**Control (*n *= 16)**	***P*‐value**
**Unadjusted**			**0.185**
HRR_1min_	−22 ± 10	−29 ± 10	0.024*
HRR_2min_	−28 ± 12	−36 ± 11	0.024*
HRR_3min_	−30 ± 13	−39 ± 13	0.043*
HRR_4min_	−31 ± 14	−42 ± 12	0.019*
HRR_5min_	−31 ± 14	−43 ± 13	0.012*
**Adjusted for %Pred 6MWT distance**			**0.202**
HRR_1min_	−22 ± 9	−28 ± 9	0.029*
HRR_2min_	−28 ± 10	−35 ± 10	0.027*
HRR_3min_	−31 ± 11	−37 ± 11	0.056
HRR_4min_	−32 ± 12	−41 ± 12	0.024*
HRR_5min_	−32 ± 12	−42 ± 12	0.013*
**Adjusted for resting HR**			**0.228**
HRR_1min_	−22 ± 9	−28 ± 9	0.031*
HRR_2min_	−28 ± 11	−36 ± 11	0.033*
HRR_3min_	−30 ± 13	−38 ± 13	0.063
HRR_4min_	−32 ± 13	−41 ± 13	0.028*
HRR_5min_	−32 ± 13	−42 ± 13	0.018*
**Adjusted for BMI**			**0.206**
HRR_1min_	−22 ± 10	−29 ± 10	0.027*
HRR_2min_	−28 ± 11	−36 ± 11	0.027*
HRR_3min_	−30 ± 13	−38 ± 13	0.050*
HRR_4min_	−31 ± 14	−42 ± 14	0.023*
HRR_5min_	−32 ± 14	−42 ± 14	0.014*
**Adjusted for age**			**0.224**
HRR_1min_	−22 ± 10	−29 ± 10	0.032*
HRR_2min_	−28 ± 12	−36 ± 12	0.035*
HRR_3min_	−30 ± 13	−38 ± 14	0.054
HRR_4min_	−31 ± 14	−41 ± 14	0.026*
HRR_5min_	−32 ± 14	−42 ± 14	0.017*

Abbreviations: BMI, body mass index; HR, heart rate; HRR_1min,_ HRR_2min_, etc., the difference in HR immediately post‐6MWT compared with each minute of recovery; SARS‐CoV‐2, severe acute respiratory syndrome coronavirus 2; 6MWT, 6‐minute walk test; %Pred 6MWT distance, distance travelled during the 6MWT normalized to a reference sample using predictive equations provided by Casanova et al. (2011). *Note*. The *P*‐value shown in bold is the omnibus test statistic for MANOVA/MANCOVA.

* *P* ≤ 0.05 between SARS‐CoV‐2 and control groups.

### 3.4 Differences observed in %Pred 6MWT distance and HR responses between control subjects, symptomatic and asymptomatic SARS‐CoV‐2 participants

When stratifying our SARS‐CoV‐2 sample by specific symptoms reported at the time of testing (i.e., symptomatic and asymptomatic), group differences were detected for %Pred 6MWT distance, ΔHR rest to post‐6MWT and HRR_1min_ between control subjects, asymptomatic and symptomatic SARS‐CoV‐2 participants (Table 3). The %Pred 6MWT distance was lower among participants reporting shortness of breath in comparison to asymptomatic SARS‐CoV‐2 participants (*P* = 0.008) and control subjects (*P* = 0.012). Likewise, the %Pred 6MWT distance was lower among those reporting joint/muscle aches in comparison to the asymptomatic SARS‐CoV‐2 group (*P* = 0.045) and control subjects (*P* = 0.043). The ΔHR from rest to post‐6MWT was also attenuated for SARS‐CoV‐2 participants reporting shortness of breath compared with the asymptomatic group (*P* = 0.026) and control subjects (*P* = 0.002). Compared with control subjects, the HRR_1min_ was delayed for SARS‐CoV‐2 participants reporting shortness of breath (*P* = 0.008), joint/muscle aches (*P* = 0.015), fatigue (*P* = 0.013) and loss of taste/smell (*P* = 0.037). Moreover, differences in HRR_1min_ trended toward significance (*P* = 0.063), with SARS‐CoV‐2 participants who reported cognitive impairment at the time of testing exhibiting a more delayed HRR compared with control subjects. There were no differences between groups when stratified based on ‘cough’ for the %Pred 6MWT distance, ΔHR from rest to post‐6MWT, and HRR_1min_. Symptoms reported in fewer than three participants at the time of testing were not examined.

**TABLE 3:** Group differences in %Pred 6MWT distance and heart rate responses between control subjects, asymptomatic and symptomatic SARS‐CoV‐2 participants

**Parameter**	**SARS‐CoV‐2, symptomatic**	**SARS‐CoV‐2, asymptomatic**	**Control**	***P*‐value**
**Shortness of breath**	***n* = 5**	***n *= 23**	***n *= 16**	
%Pred 6MWT distance, %	79 ± 15^††^	101 ± 15**	101 ± 11*	0.008**
ΔHR rest to post‐6MWT, beats/min	+32 ± 17^†^	+56 ± 18*	+65 ± 18**	0.003**
HRR_1min_, beats/min	−13 ± 9	−23 ± 10	−29 ± 10**	0.009**
**Joint/muscle aches**	***n* = 4**	***n *= 24**	***n *= 16**	
%Pred 6MWT distance, %	80 ± 19^†^	100 ± 16*	101 ± 11*	0.044*
ΔHR rest to post‐6MWT, beats/min	+44 ± 32	+53 ± 18	+65 ± 18	0.068
HRR_1min_, beats/min	−13 ± 12	−23 ± 9	−29 ± 10*	0.014*
**Fatigue**	***n* = 3**	***n *= 25**	***n *= 16**	
%Pred 6MWT distance, %	87 ± 24	98 ± 16	101 ± 11	0.359
ΔHR rest to post‐6MWT, beats/min	+40 ± 24	+53 ± 20	+65 ± 18	0.050*
HRR_1min_, beats/min	−11 ± 6	−23 ± 10	−29 ± 10*	0.011*
**Cough**	***n* = 4**	***n *= 24**	***n *= 16**	
%Pred 6MWT distance, %	90 ± 15	98 ± 18	101 ± 11	0.493
ΔHR rest to post‐6MWT, beats/min	+45 ± 28	+53 ± 19	+65 ± 18	0.076
HRR_1min_, beats/min	−19 ± 11	−22 ± 10	−29 ± 10	0.071
**Loss of taste/smell**	***n* = 10**	***n *= 18**	***n *= 16**	
%Pred 6MWT distance, %	89 ± 20	102 ± 14	101 ± 11	0.092
ΔHR rest to post‐6MWT, beats/min	+47 ± 23	+54 ± 19	+65 ± 18	0.060
HRR_1min_, beats/min	−19 ± 13	−23 ± 8	−29 ± 10*	0.041*
**Cognitive impairment**	***n* = 3**	***n *= 25**	***n *= 16**	
%Pred 6MWT distance, %	84 ± 19	99 ± 17	101 ± 11	0.229
ΔHR rest to post‐6MWT, beats/min	+64 ± 32	+50 ± 19	+65 ± 18^†^	0.046*
HRR_1min_, beats/min	−14 ± 12	−22 ± 10	−29 ± 10	0.034*

Abbreviations: HR, heart rate; HRR_1min_, the difference in HR immediately post‐6MWT compared with the initial minute of recovery; 6MWT, 6‐minute walk test; %Pred 6MWT distance, distance travelled during the 6MWT normalized to a reference sample using predictive equations provided by Casanova et al. (2011); SARS‐CoV‐2, severe acute respiratory syndrome coronavirus 2. *Note*: Significant differences from symptomatic groups are indicated as follows

* *P* ≤ 0.05

** *P* ≤ 0.01. Significant differences from asymptomatic groups are indicated as follows

^†^ *P* ≤ 0.05

^††^ *P* ≤ 0.01.

### 3.5 Associations between %Pred 6MWT distance and HR responses among SARS‐CoV‐2 participants

For the SARS‐CoV‐2 group, having a lower %Pred 6MWT distance was associated with a lower percentage of GLI reference for FEV_1_ (*r* = 0.459; *P* = 0.016), DLCO (*r *= 0.451; *P* = 0.018) and VA (*r* = 0.439; *P* = 0.022), more days reported for fever (*r* = −0.432; *P* = 0.022), shortness of breath (*r* = −0.534; *P* = 0.003) and headache (*r* = −0.378; *P* = 0.048) at illness onset. An attenuated ΔHR from rest to post‐6MWT was associated with more days reported for shortness of breath (*r* = −0.463; *P* = 0.013). Lastly, a delayed HRR_1min_ was associated with a lower percentage of GLI reference for DLCO (*r* = 0.437; *P* = 0.023). Significant associations between the other pulmonary function parameters or symptoms for %Pred 6MWT distance, ΔHR for rest to post‐6MWT, or HRR_1min_ were not observed.

### 3.6 Between‐group differences in pulmonary function testing

Among the SARS‐CoV‐2 group, FRC was below the LLN in nine of 28 participants (32%). Likewise, TLC was below the LLN in five (18%), VC in four (14%), DLCO in two (7%), VA in one (3%), RV in one (4%) and FVC in one (4%) of 28 SARS‐CoV‐2 participants. This compares to only one of 16 (6%) control subjects exhibiting values below the LLN for FEV_1_, TLC, VC and IC. SARS‐CoV‐2 was associated with having an FRC reduced below the LLN (*P* = 0.0164).

**FIGURE 3:**
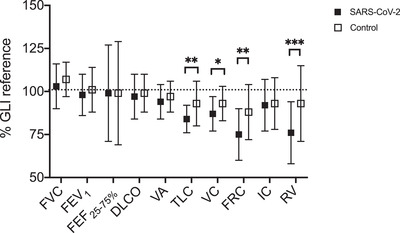
Pulmonary function test measures reported as a percentage of Global Lung Initiative (GLI) reference. Abbreviations: DLCO, diffusing capacity for carbon monoxide; FEF_25–75%_, forced expiratory flow rate between 25 and 75% of expired vital capacity; FEV_1_, forced expiratory volume in 1 s; FRC, functional residual capacity; FVC, forced vital capacity; IC, inspiratory capacity; RV, residual volume; TLC, total lung capacity; VA, alveolar volume; VC, vital capacity. For the severe acute respiratory syndrome coronavirus 2 (SARS‐CoV‐2) group (*n* = 29), one participant was excluded from analyses for spirometry measures owing to an inability to meet repeatability criteria for FVC and FEV_1_, one was excluded from DLCO analyses owing to an inability to attain the acceptable threshold for inspiratory volume, and one was excluded from lung volume analyses owing to an inability to perform a complete N_2_ washout test. For control subjects (*n* = 16), one subject was excluded from DLCO analyses owing to inability to attain acceptable threshold for inspiratory volume. Non‐normally distributed data (FVC for SARS‐CoV‐2 group and FRC and RV for control subjects) are displayed as the median ± interquartile range. All other data are normally distributed and displayed as the mean ± SD. Significant differences between groups are indicated as follows: ^*^ *P* ≤ 0.05; ^**^ *P* ≤ 0.01; ^***^ *P* ≤ 0.001

There were no differences between SARS‐CoV‐2 participants and control subjects for FVC (103 ± 13 vs. 107 ± 10%; *P* = 0.055), FEV_1_ (98 ± 12 vs. 100 ± 13%; *P* = 0.542), FEF_25–75%_ (99 ± 28 vs. 99 ± 30%; *P* = 0.961), DLCO (97 ± 13 vs. 99 ± 11%; *P* = 0.533), VA (94 ± 10 vs. 99 ± 8%; *P* = 0.178) or IC (92 ± 15 vs. 93 ± 15%; *P* = 0.815). Compared with control subjects, SARS‐CoV‐2 participants had reduced TLC (84 ± 8 vs. 93 ± 13%; *P* = 0.006), VC (87 ± 10 vs. 93 ± 10%; *P* = 0.040), FRC (75 ± 16 vs. 88 ± 16%; *P* = 0.006) and RV (76 ± 18 vs. 93 ± 22%; *P* = 0.001). Between‐group differences in pulmonary function test measures normalized to GLI reference values are shown in Figure 3.

## 4 DISCUSSION

Herein, we report differences in chronotropic responses to and recovery from a standardized 6MWT in women ∼3 months after the onset of mild‐to‐moderate SARS‐CoV‐2 infection in comparison to control subjects of similar age and BMI. Women reporting shortness of breath at the time of testing exhibited an attenuated increase in HR immediately post‐6MWT compared with control subjects and SARS‐CoV‐2 participants not actively experiencing this symptom. Additionally, HRR was delayed to a greater extent in those who reported shortness of breath, joint/muscle aches, fatigue or loss of taste/smell at the time of testing compared with control subjects. A lower %Pred 6MWT distance was also observed among women reporting shortness of breath or joint/muscle aches compared with control subjects and SARS‐CoV‐2 participants not actively experiencing these symptoms. Although delayed HRR was associated with a lower DLCO, interestingly, no associations were found between chronotropic responses to the 6MWT and lung volumes despite a considerable proportion of our SARS‐CoV‐2 cohort demonstrating values below the LLNs. Taken together, our data highlight a need for further investigation and for implementation of targeted physical rehabilitation programmes to manage the functional consequences of persistent cardiopulmonary sequelae in women exhibiting persistent shortness of breath, joint/muscle aches, fatigue and/or loss of taste/smell attributed to post‐acute COVID‐19 syndrome.

Differences in chronotropic responses to and recovery from 6MWT were observed between the SARS‐CoV‐2 group and control subjects. Our observations revealed that despite a similar distance travelled and subjective rating of perceived effort between groups, postexercise HR and HRR were attenuated in SARS‐CoV‐2 compared with control subjects. A modest increase in HR (≤ +20 beats/min) and/or attenuated HRR_1min_ (≤ −13 beats/min) to the 6MWT are strong independent predictors of disease progression in respiratory pathologies characterized by restricted lung volumes and impaired gas exchange, such as idiopathic pulmonary fibrosis (Holland et al., 2013; Swigris et al., 2009). Interestingly, we observed four of 28 (14%) participants recovering from SARS‐CoV‐2 exhibiting abnormal HR responses at or below these thresholds, while 11 of 28 (39%) demonstrated HR responses below the fifth percentile for control values. An attenuated increase and/or delayed HRR was associated with a lower DLCO and greater number of days experiencing shortness of breath at illness onset. A more delayed HRR was also observed in women actively experiencing shortness of breath, joint/muscle aches, fatigue or loss of taste/smell compared with control subjects.

Although the 6MWT distance did not differ between control subjects and the entire SARS‐CoV‐2 cohort (*n* = 29), group differences emerged when SARS‐CoV‐2 participants were stratified by symptoms reported at the time of testing. Those reporting shortness of breath or joint/muscle aches achieved a lower %Pred 6MWT distance compared with control subjects and SARS‐CoV‐2 participants not actively experiencing these symptoms. Having a lower %Pred 6MWT was also associated with a lower FEV_1_, DLCO, VA, and greater number of days experiencing fever, shortness of breath and headache at illness onset. Taken together, these findings suggest that submaximal exercise capacity is compromised in women actively experiencing certain symptoms after acute COVID‐19. It is also possible that shortness of breath and joint/muscle aches reported several months into recovery might reflect persistent pulmonary abnormalities (i.e., reduced DLCO and VA) impairing gas exchange and tolerance to submaximal physical activity in this population.

Other accounts have provided evidence supporting impaired chronotropic responses to maximal exercise in individuals recovering from COVID‐19 (Dorelli et al., 2021; Szekely et al., 2021). Chronotropic incompetence (defined as failure to achieve ≥ 80% HR reserve) during a maximal cardiopulmonary exercise test was observed in 75% of individuals ∼3 months into recovery from mild‐to‐critical SARS‐CoV‐2 illness compared with 8% of control subjects (Szekely et al., 2021). These changes were accompanied by higher right atrial pressures at rest along with reduced left ventricular end‐diastolic volume and left ventricular ejection fraction during exercise (Szekely et al., 2021). Additionally, a delayed HRR during the initial minute after maximal graded exercise has been documented in previously hospitalized SARS‐CoV‐2 patients exhibiting exercise ventilatory inefficiency (minute ventilation/carbon dioxide production slope above upper limits of normal) in comparison to those with normal ventilatory efficiency slopes (Dorelli et al., 2021).

Haemodynamic and ventilatory irregularities observed in some individuals recovering from COVID‐19 are characteristic of the physiological consequences of pulmonary vascular remodelling with pulmonary arterial hypertension (Querejeta Roca et al., 2015; Weatherald et al., 2018; Wu et al., 2017). Indeed, severe SARS‐CoV‐2 infection, via either direct viral infiltration of pulmonary capillary endothelial cells and/or resultant inflammation, has been associated with microvascular dysfunction and subsequent perfusion impairments resulting from increased physiological dead space (Cascino et al., 2021). It is possible that a higher right ventricular afterload with elevated pulmonary arterial pressures could lead to sympathoexcitation, thereby altering the sympathovagal balance during recovery from SARS‐CoV‐2 (Wu et al., 2017). Although our data cannot confirm whether this assumption applies to women recovering from mild‐to‐moderate SARS‐CoV‐2 illness, severe abnormalities in DLCO and VA observed in several (*n* = 2) of our participants and the relationship of DLCO to HRR and %Pred 6MWT distance are consistent with this proposed aetiology of impaired chronotropic responses to submaximal physical exertion. However, additional investigations are warranted to clarify the exact mechanisms contributing to these observed abnormalities.

As an alternative explanation for impaired HR responses among the present SARS‐CoV‐2 cohort, accumulating evidence suggests that SARS‐CoV‐2, similar to other coronaviruses, has neurotropic properties (Jarrahi et al., 2020). Case reports have revealed the development of phrenic nerve paralysis in COVID‐19 patients without computed tomographic evidence of lung damage (Maurier et al., 2020). Additional reports of COVID‐19 patients developing postural tachycardia syndrome (Kanjwal et al., 2020; Miglis et al., 2020; Umapathi et al., 2020) provide further evidence for impaired cardiac vagal modulation (Jacob et al., 2019), possibly related to either direct viral attack of the nervous system or ensuing autoimmune responses following SARS‐CoV‐2 infection that are associated with autonomic disorders (Dani et al., 2021). Collectively, such findings indicate possible infestation and impairment of the phrenic and vagal nerves in COVID‐19 patients, which might account for the observed alterations in chronotropic responses to the 6MWT.

Respiratory muscle weakness of unknown aetiology has also been reported in COVID‐19 patients (Farr et al., 2020), which might offer an explanation for abnormal lung volumes observed in our cohort (Hart et al., 2002). Consistent with previous accounts demonstrating abnormal lung volumes in individuals recovering from moderate‐to‐critical COVID‐19 (Ekbom et al., 2021; González et al., 2021; Huang et al., 2021; Smet et al., 2021), we observed FRC, TLC, VC and RV that were below clinically relevant thresholds in several of our participants recovering from mild‐to‐moderate illness. However, the proportion of SARS‐CoV‐2 participants demonstrating abnormal lung volumes in our study was lower than that found in accounts including hospitalized cases of greater severity (Ekbom et al., 2021; González et al., 2021; Huang et al., 2021; Smet et al., 2021). Further examination of the mechanisms underlying reduced lung volumes (i.e., fibrotic/inflammatory changes to the pulmonary interstitium, respiratory muscle weakness attributed to neuromuscular manifestations of the virus, and microcirculatory changes leading to atrophy of type II myofibres; Farr et al., 2020), via diagnostic imaging and/or tests of respiratory muscle strength, is needed in individuals experiencing post‐acute COVID‐19 syndrome.

Reduced lung volumes, particularly FRC, are suggestive of decreased lung compliance, the potential consequences of which are limited exercise tolerance owing to greater airway resistance and work of breathing (Lutfi, 2017). However, exercise tolerance was largely preserved in our SARS‐CoV‐2 cohort, as indicated by similar distances covered during the 6MWT compared with control subjects. Furthermore, we observed no associations between %Pred 6MWT distance and lung volumes. Given the relatively low pressures presumably generated by the respiratory muscles during the 6MWT, it is highly unlikely that O_2_ delivery would be compromised owing to increased respiratory muscle work (Romer & Polkey, 2008). Therefore, the relationship between %Pred 6MWT and lung volumes would be expected. Yet, our observations cannot eliminate the possibility that the capacity to perform higher‐intensity exercise would be impaired in women recovering from mild‐to‐moderate illness, especially given that peak aerobic capacity appears to be reduced during recovery from mild‐to‐critical COVID‐19 in comparison to matched control subjects (Singh et al., 2021; Szekely et al., 2021). Additional work is needed to clarify whether relationships exist between reduced lung volumes and exercise tolerance at higher relative work intensities among individuals recovering from COVID‐19.

By adopting a case–control matched design, we have greater assurance in attributing the present chronotropic responses to the 6MWT to post‐acute COVID‐19 syndrome rather than underlying irregularities related to ageing or obesity. Nevertheless, given the retrospective nature of this work, we cannot disregard the possibility of undiagnosed pulmonary abnormalities and/or autonomic dysfunction that existed before a SARS‐CoV‐2 infection. Additionally, it is possible that individuals included in the control group might have had an asymptomatic SARS‐CoV‐2 infection and/or a previous false‐negative diagnostic test for SARS‐CoV‐2. Therefore, we cannot conclude with absolute certainty that the abnormalities observed were attributable to the effects of the SARS‐CoV‐2 virus alone.

Furthermore, future investigations should consider appropriately characterizing menopausal status (e.g., years since menopause, early‐ or late‐menopausal transition) and use of hormonal replacement therapies, because women in the late‐menopausal transition and menopause have a greater prevalence of restrictive lung abnormalities (Hong et al., 2021) that are partly reversed by the administration of combined hormonal therapies (Cevrioglu et al., 2004). Although we did not control for menopausal status in the present investigation, the proportion of women exceeding the average age of menopause (51 years) was similar in the SAR‐CoV‐2 (79%) and control (81%) groups. It is also possible that hormonal changes during the menopausal transition might increase susceptibility to chronic symptoms after a SARS‐CoV‐2 infection, considering that the majority of patients seeking treatment for post‐acute COVID‐19 syndrome have been women around the ages of 40–45 years old (Davido et al., 2020; Vanichkachorn et al., 2021). Future work should investigate whether altered cardiopulmonary responses to exercise similar to those observed in our account exist with recovery from COVID‐19 in men and younger (premenopausal) women.

### 4.1 Conclusions

Recent reports suggest that women are more likely to seek treatment for post‐acute COVID‐19 syndrome and to exhibit diffusion impairments for months after a SARS‐CoV‐2 infection. Nonetheless, little is known regarding the influence of such persistent cardiopulmonary abnormalities on physical functioning. Our findings provide evidence for marked differences in chronotropic responses to and recovery from a 6MWT in women several months after acute SARS‐CoV‐2 infection. Notably, women reporting specific symptoms at the time of testing had greater impairments compared with control subjects and SARS‐CoV‐2 participants not actively experiencing these symptoms. Furthermore, although DLCO was below the LLN in only two of 28 participants recovering from SARS‐CoV‐2, a lower DLCO was associated with a lower %Pred 6MWT distance and more pronounced delay in HRR among this group. Such alterations have potential to constrain not only exercise tolerance but also participation in free‐living physical activity in women during post‐acute recovery from COVID‐19.

## COMPETING INTERESTS

None declared.

## AUTHOR CONTRIBUTIONS

All experiments were performed in the Human Performance Laboratory at the Indiana University School of Public Health – Bloomington. M.N.B. conducted experimental testing, completed analyses and drafted the original manuscript. S.J.C. obtained funding and provided supervision of experimental testing and analyses. Both authors conceptualized the study design, provided revisions to manuscript drafts, approved of the final version of the manuscript and agree to be accountable for all aspects of the work in ensuring that questions related to the accuracy or integrity of any part of the work are appropriately investigated and resolved. Both persons designated as authors qualify for authorship, and both those who qualify for authorship are listed.

## Supporting information


Statistical Summary Document

## Data Availability

The data that support the findings of this study are available from the corresponding author upon reasonable request.

## References

[eph13116-bib-0001] ATS Committee on Proficiency Standards for Clinical Pulmonary Function Laboratories (2002) ATS statement: Guidelines for the six‐minute walk test. American Journal of Respiratory and Critical Care Medicine, 166(1), 111–117. 10.1164/ajrccm.166.1.at1102] 12091180

[eph13116-bib-0002] Bellan, M. , Soddu, D. , Balbo, P. E. , Baricich, A. , Zeppegno, P. , Avanzi, G. C. , Baldon, G. , Bartolomei, G. , Battaglia, M. , Battistini, S. , Binda, V. , Borg, M. , Cantaluppi, V. , Castello, L. M. , Clivati, E. , Cisari, C. , Costanzo, M. , Croce, A. , Cuneo, D. … Pirisi, M. (2021). Respiratory and psychophysical sequelae among patients with COVID‐19 four months after hospital discharge. JAMA Network Open, 4(1), e2036142. 10.1001/jamanetworkopen.2020.36142 33502487 PMC7841464

[eph13116-bib-1001] Borg, G. , (1998). Borg's perceived exertion and pain scales. Human Kinetics.

[eph13116-bib-0003] Casanova, C. , Celli, B. R. , Barria, P. , Casas, A. , Cote, C. , de Torres, J. P. , Jardim, J. , Lopez, M. V. , Marin, J. M. , Montes de Oca, M. , Pinto‐Plata, V. , & Aguirre‐Jaime, A. (2011). The 6‐min walk distance in healthy subjects: Reference standards from seven countries. European Respiratory Journal, 37(1), 150–156. 10.1183/09031936.00194909 20525717

[eph13116-bib-0004] Cascino, T. M. , Desai, A. A. , & Kanthi, Y. (2021). At a crossroads: Coronavirus disease 2019 recovery and the risk of pulmonary vascular disease. Current Opinion in Pulmonary Medicine, 27(5), 342–349. 10.1097/mcp.0000000000000792 34127622 PMC8373709

[eph13116-bib-0005] Cevrioglu, A. S. , Fidan, F. , Unlu, M. , Yilmazer, M. , Orman, A. , Fenkci, I. V. , & Serteser, M. (2004). The effects of hormone therapy on pulmonary function tests in postmenopausal women. Maturitas, 49(3), 221–227. 10.1016/j.maturitas.2004.01.009 15488350

[eph13116-bib-0006] COVID‐19 Treatment Guidelines Panel . Coronavirus disease (2019) (COVID‐19) treatment guidelines. National Institutes of Health. https://www.covid19treatmentguidelines.nih.gov/ 34003615

[eph13116-bib-0007] Dani, M. , Dirksen, A. , Taraborrelli, P. , Torocastro, M. , Panagopoulos, D. , Sutton, R. , & Lim, P. B. (2021). Autonomic dysfunction in ‘long COVID’: Rationale, physiology and management strategies. Clinical Medicine (London, England), 21(1), e63–e67. 10.7861/clinmed.2020-0896 PMC7850225 33243837

[eph13116-bib-0008] Davido, B. , Seang, S. , Tubiana, R. , & de Truchis, P. (2020). Post‐COVID‐19 chronic symptoms: A postinfectious entity? Clinical Microbiology and Infection, 26(11), 1448–1449. 10.1016/j.cmi.2020.07.028 32712242 PMC7376333

[eph13116-bib-0009] Dorelli, G. , Braggio, M. , Gabbiani, D. , Busti, F. , Caminati, M. , Senna, G. , Girelli, D. , Laveneziana, P. , Ferrari, M. , Sartori, G. , Carbonare, L. D. , & Crisafulli, E. ; the Respicovid Study Ivestigators . (2021). Importance of cardiopulmonary exercise testing amongst subjects recovering from COVID‐19. Diagnostics (Basel), 11(3), 507. 10.3390/diagnostics11030507 33809260 PMC7998697

[eph13116-bib-0010] Ekbom, E. , Frithiof, R. , Emilsson, O. , Larson, I. , Lipcsey, M. , Rubertsson, S. , Wallin, E. , Janson, C. , Hultström, M. , & Malinovschi, A. (2021). Impaired diffusing capacity for carbon monoxide is common in critically ill Covid‐19 patients at four months post‐discharge. Respiratory Medicine, 182, 106394. 10.1016/j.rmed.2021.106394 33901787 PMC8047337

[eph13116-bib-0011] Farr, E. , Wolfe, A. R. , Deshmukh, S. , Rydberg, L. , Soriano, R. , Walter, J. M. , Boon, A. J. , Wolfe, L. F. , & Franz, C. K. (2020). Short of breath for the long haul: Diaphragm muscle dysfunction in survivors of severe COVID‐19 as determined by neuromuscular ultrasound. *medRxiv* , 2020.2012.2010.20244509. 10.1101/2020.12.10.20244509 medRxiv

[eph13116-bib-0012] González, J. , Benítez, I. D. , Carmona, P. , Santisteve, S. , Monge, A. , Moncusí‐Moix, A. , Gort‐Paniello, C. , Pinilla, L. , Carratalá, A. , Zuil, M. , Ferrer, R. , Ceccato, A. , Fernández, L. , Motos, A. , Riera, J. , Menéndez, R. , Garcia‐Gasulla, D. , Peñuelas, O. , Bermejo‐Martin, J. F. , & Barbé, F. (2021). Pulmonary function and radiologic features in survivors of critical COVID‐19: A 3‐month prospective cohort. Chest, 160(1), 187–198. 10.1016/j.chest.2021.02.062 33676998 PMC7930807

[eph13116-bib-0013] Graham, B. L. , Brusasco, V. , Burgos, F. , Cooper, B. G. , Jensen, R. , Kendrick, A. , MacIntyre, N. R. , Thompson, B. R. , & Wanger, J. (2017). 2017 ERS/ATS standards for single‐breath carbon monoxide uptake in the lung. European Respiratory Journal, 49(1), 1600016. 10.1183/13993003.00016-2016 28049168

[eph13116-bib-0014] Graham, B. L. , Steenbruggen, I. , Miller, M. R. , Barjaktarevic, I. Z. , Cooper, B. G. , Hall, G. L. , Hallstrand, T. S. , Kaminsky, D. A. , McCarthy, K. , McCormack, M. C. , Oropez, C. E. , Rosenfeld, M. , Stanojevic, S. , Swanney, M. P. , & Thompson, B. R. (2019). Standardization of spirometry 2019 update. An official American Thoracic Society and European Respiratory Society technical statement. American Journal of Respiratory and Critical Care Medicine, 200(8), e70–e88. 10.1164/rccm.201908-1590ST 31613151 PMC6794117

[eph13116-bib-0015] Hart, N. , Cramer, D. , Ward, S. P. , Nickol, A. H. , Moxham, J. , Polkey, M. I. , & Pride, N. B. (2002). Effect of pattern and severity of respiratory muscle weakness on carbon monoxide gas transfer and lung volumes. European Respiratory Journal, 20(4), 996–1002. 10.1183/09031936.00.00286702 12412695

[eph13116-bib-0016] Holland, A. E. , Hill, C. J. , Glaspole, I. , Goh, N. , Dowman, L. , & McDonald, C. F. (2013). Impaired chronotropic response to 6‐min walk test and reduced survival in interstitial lung disease. Respiratory Medicine, 107(7), 1066–1072. 10.1016/j.rmed.2013.04.002 23669412

[eph13116-bib-0017] Hong, Y. S. , Park, H. Y. , Chang, Y. , Jang, E. H. , Zhao, D. , Kim, S. , Guallar, E. , Kim, H. , Cho, J. , & Ryu, S. (2021). Stages of menopause and abnormal lung function: A cross‐sectional study of middle‐aged women. Menopause, 28(7), 811–818. 10.1097/gme.0000000000001779 33828036

[eph13116-bib-0018] Huang, C. , Huang, L. , Wang, Y. , Li, X. , Ren, L. , Gu, X. , Kang, L. , Guo, L. , Liu, M. , Zhou, X. , Luo, J. , Huang, Z. , Tu, S. , Zhao, Y. , Chen, L. , Xu, D. , Li, Y. , Li, C. , Peng, L. , … Cao, B. (2021). 6‐month consequences of COVID‐19 in patients discharged from hospital: A cohort study. Lancet, 397(10270), 220–232. 10.1016/s0140-6736(20)32656-8 33428867 PMC7833295

[eph13116-bib-0019] Jacob, G. , Diedrich, L. , Sato, K. , Brychta, R. J. , Raj, S. R. , Robertson, D. , Biaggioni, I. , &., & Diedrich, A. (2019). Vagal and sympathetic function in neuropathic postural tachycardia syndrome. Hypertension, 73(5), 1087–1096. 10.1161/hypertensionaha.118.11803 30879357 PMC6592426

[eph13116-bib-0020] Jarrahi, A. , Ahluwalia, M. , Khodadadi, H. , da Silva Lopes Salles, E. , Kolhe, R. , Hess, D. C. , Vale, F. , Kumar, M. , Baban, B. , Vaibhav, K. , & Dhandapani, K. M. (2020). Neurological consequences of COVID‐19: What have we learned and where do we go from here? Journal of Neuroinflammation, 17(1), 286. 10.1186/s12974-020-01957-4 32998763 PMC7525232

[eph13116-bib-0021] Kanjwal, K. , Jamal, S. , Kichloo, A. , & Grubb, B. P. (2020). New‐onset postural orthostatic tachycardia syndrome following coronavirus disease 2019 infection. Journal of Innovations in Cardiac Rhythm Management, 11(11), 4302–4304. 10.19102/icrm.2020.111102 33262898 PMC7685310

[eph13116-bib-0022] Lancaster, L. H. (2018). Utility of the six‐minute walk test in patients with idiopathic pulmonary fibrosis. Multidisciplinary Respiratory Medicine, 13, 45. 10.1186/s40248-018-0158-z 30559965 PMC6291931

[eph13116-bib-0023] Lutfi, M. F. (2017). The physiological basis and clinical significance of lung volume measurements. Multidisciplinary Respiratory Medicine, 12, 3. 10.1186/s40248-017-0084-5 28194273 PMC5299792

[eph13116-bib-0024] Maurier, F. , Godbert, B. , & Perrin, J. (2020). Respiratory distress in SARS‐CoV‐2 without lung damage: Phrenic paralysis should be considered in COVID‐19 infection. European Journal of Case Reports in Internal Medicine, 7(6), 001728. 10.12890/2020_001728 32523929 PMC7279902

[eph13116-bib-0025] Miglis, M. G. , Prieto, T. , Shaik, R. , Muppidi, S. , Sinn, D. I. , & Jaradeh, S. (2020). A case report of postural tachycardia syndrome after COVID‐19. Clinical Autonomic Research, 30(5), 449–451. 10.1007/s10286-020-00727-9 32880754 PMC7471493

[eph13116-bib-0026] Morita, A. A. , Silva, L. K. O. , Bisca, G. W. , Oliveira, J. M. , Hernandes, N. A. , Pitta, F. , & Furlanetto, K. C. (2018). Heart rate recovery, physical activity level, and functional status in subjects with COPD. Respiratory Care, 63(8), 1002–1008. 10.4187/respcare.05918 29765005

[eph13116-bib-0027] Nalbandian, A. , Sehgal, K. , Gupta, A. , Madhavan, M. V. , McGroder, C. , Stevens, J. S. , Cook, J. R. , Nordvig, A. S. , Shalev, D. , Sehrawat, T. S. , Ahluwalia, N. , Bikdeli, B. , Dietz, D. , Der‐Nigoghossian, C. , Liyanage‐Don, N. , Rosner, G. F. , Bernstein, E. J. , Mohan, S. , Beckley, A. A. , … Wan, E. Y. (2021). Post‐acute COVID‐19 syndrome. Nature Medicine, 27(4), 601–615. 10.1038/s41591-021-01283-z PMC8893149 33753937

[eph13116-bib-0028] Orzes, N. , Pini, L. , Levi, G. , Uccelli, S. , Cettolo, F. , & Tantucci, C. (2021). A prospective evaluation of lung function at three and six months in patients with previous SARS‐COV‐2 pneumonia. Respiratory Medicine, 186, 106541. 10.1016/j.rmed.2021.106541 34280885 PMC8272067

[eph13116-bib-0029] Peckham, H. , de Gruijter, N. M. , Raine, C. , Radziszewska, A. , Ciurtin, C. , Wedderburn, L. R. , Rosser, E. C. , Webb, K. , & Deakin, C. T. (2020). Male sex identified by global COVID‐19 meta‐analysis as a risk factor for death and ITU admission. Nature Communications, 11(1), 6317. 10.1038/s41467-020-19741-6 PMC7726563 33298944

[eph13116-bib-0030] Quanjer, P. H. , Stanojevic, S. , Cole, T. J. , Baur, X. , Hall, G. L. , Culver, B. H. , Enright, P. L. , Hankinson, J. L. , Ip, M. S. M. , Zheng, J. , & Stocks, J. (2012). Multi‐ethnic reference values for spirometry for the 3–95‐yr age range: The global lung function 2012 equations. European Respiratory Journal, 40(6), 1324–1343. 10.1183/09031936.00080312 22743675 PMC3786581

[eph13116-bib-0031] Querejeta Roca, G. , Campbell, P. , Claggett, B. , Solomon, S. D. , & Shah, A. M. (2015). Right atrial function in pulmonary arterial hypertension. Circulation. Cardiovascular Imaging, 8(11), e003521. 10.1161/CIRCIMAGING.115.003521 26514759 PMC4629509

[eph13116-bib-0032] Romer, L. M. , & Polkey, M. I. (2008). Exercise‐induced respiratory muscle fatigue: Implications for performance. Journal of Applied Physiology, 104(3), 879–888. 10.1152/japplphysiol.01157.2007 18096752

[eph13116-bib-0033] Safont , B. , Tarraso, J. , Rodriguez‐Borja, E. , Fernández‐Fabrellas, E. , Sancho‐Chust, J. N. , Molina, V. , Lopez‐Ramirez, C. , Lope‐Martinez, A. , Cabanes, L. , Andreu, A. L. , Herrera, S. , Lahosa, C. , Ros, J. A. , Rodriguez‐Hermosa, J. L. , Soriano, J. B. , Moret‐Tatay, I. , Carbonell‐Asins, J. A. , Mulet, A. , & Signes‐Costa, J. (2021). Lung function, radiological findings and biomarkers of fibrogenesis in a cohort of COVID‐19 patients six months after hospital discharge. Archivos de Bronconeumologia. Advance online publication. 10.1016/j.arbres.2021.08.014 PMC8414844 34497426

[eph13116-bib-0034] Singh , I. , Joseph, P. , Heerdt, P. M. , Cullinan, M. , Lutchmansingh, D. D. , Gulati, M. , Possick, J. D. , Systrom, D. M. , & Waxman, A. B. (2021). Persistent exertional intolerance after covid‐19: Insights from invasive cardiopulmonary exercise testing. Chest. Advance online publication. 10.1016/j.chest.2021.08.010 PMC8354807 34389297

[eph13116-bib-0035] Smet, J. , Stylemans, D. , Hanon, S. , Ilsen, B. , Verbanck, S. , & Vanderhelst, E. (2021). Clinical status and lung function 10 weeks after severe SARS‐CoV‐2 infection. Respiratory Medicine, 176, 106276. 10.1016/j.rmed.2020.106276 33278758 PMC7701883

[eph13116-bib-0036] Swigris, J. J. , Swick, J. , Wamboldt, F. S. , Sprunger, D. , du Bois, R. , Fischer, A. , Cosgrove, G. P. , Frankel, S. K. , Fernandez‐Perez, E. R. , Kervitsky, D. , & Brown, K. K. (2009). Heart rate recovery after 6‐min walk test predicts survival in patients with idiopathic pulmonary fibrosis. Chest, 136(3), 841–848. 10.1378/chest.09-0211 19395579 PMC2775995

[eph13116-bib-0037] Szekely , Y. , Lichter, Y. , Sadon, S. , Lupu, L. , Taieb, P. , Banai, A. , Sapir, O. , Granot, Y. , Hochstadt, A. , Friedman, S. , Laufer‐Perl, M. , Banai, S. , & Topilsky, Y. (2021). Cardiorespiratory abnormalities in patients recovering from cooronavirus disease 2019. Journal of the American Society of Echocardiography, 10.1016/j.echo.2021.08.022 PMC8425293 34508837

[eph13116-bib-0038] Umapathi, T. , Poh, M. Q. W. , Fan, B. E. , Li, K. F. C. , George, J. , & Tan, J. Y. L. (2020). Acute hyperhidrosis and postural tachycardia in a COVID‐19 patient. Clinical Autonomic Research, 30(6), 571–573. 10.1007/s10286-020-00733-x 32970212 PMC7511524

[eph13116-bib-0039] Vanichkachorn, G. , Newcomb, R. , Cowl, C. T. , Murad, M. H. , Breeher, L. , Miller, S. , Trenary, M. , Neveau, D. , & Higgins, S. (2021). Post‐COVID‐19 syndrome (long haul syndrome): Description of a multidisciplinary clinic at Mayo Clinic and characteristics of the initial patient cohort. Mayo Clinic Proceedings, 96(7), 1782–1791. 10.1016/j.mayocp.2021.04.024 34218857 PMC8112396

[eph13116-bib-0040] Wanger, J. , Clausen, J. L. , Coates, A. , Pedersen, O. F. , Brusasco, V. , Burgos, F. , Casaburi, R. , Crapo, R. , Enright, P. , van der Grinten, C. P. M. , Gustafsson, P. , Hankinson, J. , Jensen, R. , Johnson, D. , MacIntyre, N. , McKay, R. , Miller, M. R. , Navajas, D. , Pellegrino, R. , & Viegi, G. (2005). Standardisation of the measurement of lung volumes. European Respiratory Journal, 26(3), 511–522. 10.1183/09031936.05.00035005 16135736

[eph13116-bib-0041] Weatherald, J. , Sattler, C. , Garcia, G. , & Laveneziana, P. (2018). Ventilatory response to exercise in cardiopulmonary disease: The role of chemosensitivity and dead space. European Respiratory Journal, 51(2), 1700860. 10.1183/13993003.00860-2017 29437936

[eph13116-bib-0042] Wu, C. , Guo, J. , Liu, H. , Pudasaini, B. , Yang, W. , Zhao, Q. , Wang, L. , & Liu, J. (2017). The correlation of decreased heart rate recovery and chronotropic incompetence with exercise capacity in idiopathic pulmonary arterial hypertension patients. BioMed Research International, 2017, 3145401. 10.1155/2017/3415401 PMC5329652 28286762

[eph13116-bib-0043] Xiong, Q. , Xu, M. , Li, J. , Liu, Y. , Zhang, J. , Xu, Y. , & Dong, W. (2021). Clinical sequelae of COVID‐19 survivors in Wuhan, China: A single‐centre longitudinal study. Clinical Microbiology and Infection, 27(1), 89–95. 10.1016/j.cmi.2020.09.023 32979574 PMC7510771

